# Traditional medicines and globalization: current and future perspectives in ethnopharmacology

**DOI:** 10.3389/fphar.2013.00092

**Published:** 2013-07-25

**Authors:** Marco Leonti, Laura Casu

**Affiliations:** ^1^Department of Biomedical Sciences, University of CagliariCagliari, Italy; ^2^Department of Life and Environmental Sciences, University of CagliariCagliari, Italy

**Keywords:** knowledge transmission, globalization, medical pluralism, global economy, traditional medicines, syncretism

## Abstract

The ethnopharmacological approach toward the understanding and appraisal of traditional and herbal medicines is characterized by the inclusions of the social as well as the natural sciences. Anthropological field-observations describing the local use of nature-derived medicines are the basis for ethnopharmacological enquiries. The multidisciplinary scientific validation of indigenous drugs is of relevance to modern societies at large and helps to sustain local health care practices. Especially with respect to therapies related to aging related, chronic and infectious diseases traditional medicines offer promising alternatives to biomedicine. Bioassays applied in ethnopharmacology represent the molecular characteristics and complexities of the disease or symptoms for which an indigenous drug is used in “traditional” medicine to variable depth and extent. One-dimensional *in vitro* approaches rarely cope with the complexity of human diseases and ignore the concept of polypharmacological synergies. The recent focus on holistic approaches and systems biology in medicinal plant research represents the trend toward the description and the understanding of complex multi-parameter systems. Ethnopharmacopoeias are non-static cultural constructs shaped by belief and knowledge systems. Intensified globalization and economic liberalism currently accelerates the interchange between local and global pharmacopoeias via international trade, television, the World Wide Web and print media. The increased infiltration of newly generated biomedical knowledge and introduction of “foreign” medicines into local pharmacopoeias leads to syncretic developments and generates a feedback loop. While modern and post-modern cultures and knowledge systems adapt and transform the global impact, they become more relevant for ethnopharmacology. Moreover, what is traditional, alternative or complementary medicine depends on the adopted historic-cultural perspective.

## INTRODUCTION TO ETHNOPHARMACOLOGY

Ethnopharmacology is a multidisciplinary field of inquiry investigating the anthropological rationale and the pharmacological basis of the medicinal use of plants, animals, fungi, micro-organisms, and minerals by human cultures. An “interdisciplinary approach is essential”… in “the attempts to present an equilibrated appraisal of the therapeutic potential of an indigenous drug” (Rivier and Bruhn, 1979). Richard Evans Schultes and Tony Swain reasoned: “Peoples whom we have chosen to consider members of less-advanced societies have consistently looked to the Plant Kingdom”…. Should we as chemists, pharmacologists, and botanists – with so many and varied means at our disposal – not take a lesson from them?” (Rivier and Bruhn, 1979). While commercial interests have always been a driving force behind the search for new therapies, the global economy is accelerating the commodification of indigenous and local knowledge (Posey, 2002). But today, in a more globalized world, the concern has matured that meanwhile “our goal should not be to freeze people in time. Instead we must find ways to ensure that in a pluralistic interconnected world all peoples may benefit from modernity without that engagement demanding the sacrifice of their ethnicity.” (Davis, 2010).

Traditional medicine and medicinal plants are frequently used in urban settings as alternatives in daily health care and self-medication against minor and chronic ailments but especially relied upon in less wealthy rural areas or times of economic crises. Common to most systems of “traditional” or local medicine is the adoption of a holistic perspective on human health. The evaluation of descriptive field-data, as well as the identification of anthropological aspects in traditional medicines, benefit from hypothesis-driven approaches. Well-defined and testable research questions in medical anthropology not only restrain from arbitrary conclusions but also consolidate and foster the bond between the social and the natural sciences. The focus of ethnopharmacology centers on the bio-cultural investigation and description of “traditional,” indigenous or local pharmacopoeias and the experimental evaluation of *materia medica* through biological test systems. The molecular characteristics and complexities of the disease or symptoms for which a medicinal agent is used in “traditional” medicine are represented to variable depth and extent in the bioassays used to corroborate the observed health claims. The ultimate goal of ethnopharmacology is contributing to evidence-based medicine, and to this aim, conclusive negative results may be as important as positive data.

## CHALLENGES IN ETHNOPHARMACOLOGY

“Traditional” systems of medicine often recommend complex herbal mixtures and multi-compound extracts. This polypharmacological approach is closer to the treatment of diseases related to multifactorial causes such as chronic and degenerative ailments, than biomedicine since here the concept of “one disease – one target – one drug” does not hold (Ulrich-Merzenich et al., 2009; Verpoorte et al., 2009). A mixture of moderately active metabolites present in an extract are potentially able to interfere and down regulate different proteins of the same signaling network leading to synergistic pharmacological effects (Wagner and Ulrich-Merzenich, 2009). In this respect it was also pointed out that reductionistic *in vitro* approaches are scarcely suited for the detection of synergistic properties or pro-drugs and how important it is to follow a more holistic *in vivo* approach either via clinical trials or animal experiments (Verpoorte et al., 2005). Since *in vivo* studies are not always feasible it has furthermore been outlined how -omics approaches and systems biology are conceptually nearer to the holistic perspective of ethnopharmacology (Verpoorte et al., 2005; Zhang et al., 2010a). Metabonomics for instance tries to measure as many metabolic factors as possible in order to asses and better understand the alteration, perturbation and functioning of complex biological systems upon determined exogenous stimuli such as medicinal plant extracts. Especially with respect to Chinese medicine (TCM) -omic techniques are increasingly gaining importance (for a review, see Buriani et al., 2012). Adopting a systems approach for the analysis of quality and safety (Wang et al., 2009) and rationalization of polypharmacological mechanisms or network pharmacology (Hopkins, 2007) exerted by complex mixtures of compounds and extracts complies best with the holistic standard of Chinese and Ayurvedic medicine (Joshi et al., 2010; Zhang et al., 2010a).

The enormous amount and diversity of therapeutic uses of traditional medicines described in literature, correlates all too well with their activities assessed in one-dimensional bioassays. Frequently exceedingly high – until the desired effect can be observed – and therapeutically questionable concentrations of extracts or natural products are applied to test systems. This is also the point where the story usually ends as only few claims observed *in vitro* can be repeated *in vivo* (Gertsch, 2009). The matching of ethnomedical indications with biological test systems is not always straightforward and many plant species and crude drugs are indicated and applied for different diseases and symptoms at the same time within one culture and even to a greater extend between different cultures (Spjut, 2005; Gyllenhaal et al., 2012). Moreover, ethnopharmacopoeias are cultural constructs and in constant exchange and transformation. Local use of medicinal plants and associated cultural healing concepts are able to co-exist with, and complement, the local commercialization and use of pharmaceuticals. Such co-existence frequently leads to syncretic developments as shown for rural indigenous communities in Mexico and Peru (Giovannini et al., 2011; Monigatti et al., 2012). Intensified globalization currently accelerates the interchange between local and global pharmacopoeias not only through international business interests but also via print media, television and the World Wide Web generating a feedback loop. The infiltration of global phytotherapeutical and biomedical knowledge into local pharmacopoeias may lead to circular argumentations in debates related to medical anthropology and drug discovery (Leonti, 2011). The global commercialization of herbal medicine and the emergence of what is called Complementary and Alternative Medicine as a social phenomenon during the past decades is also due to the perception among Western societies, that the consumption of such products would be devoid of health risks, especially in comparison to pharmaceuticals (Etkin, 2006, p. 207). However, such a perception is, for example, not compliant with the philosophy and criteria of Chinese medicine, which experimentally exploits the boundaries between toxic and non-toxic applications of herbal formulas and medicine in therapy (Wang et al., 2012a). Also, deliberate or accidental adulterations or misidentified herbal drugs constitute a continuous problem and latent health risk (Ouarghidi et al., 2012; Walker and Applequist, 2012).

In this perspective review, we highlight the historical legacy and development of ethnopharmacology, the relevance of human diet and the importance of allelochemicals. The interface between phytotherapy and drug discovery is evidenced with a discussion on bioprospecting ancient texts, phylogenetic approaches in ethnopharmacology, and evolutionary ecology. Through a discourse touching on aspects of knowledge transmission, integration of medicinal systems, globalization, and medicinal pluralism we tag economy and commercialism as a driving force in ethnopharmacology.

## THE GEARWHEEL OF ETHNOPHARMACOLOGY

In the introduction to the chapter of “Pharmacohistoria” to his fundamental work “Handbuch der Pharmakognosie” (Handbook of Pharmacognosy) Tschirch (1910, p. 446) quotes a colleague: “…in its true sense no discovery is really independent, each [discovery] is conditioned and most often also evocated by the actual spirit of its time – in any case, however, primed by previous works.” In the following I shall give some historical sketches and examples of how this quote may be interpreted.

Ergot, for example, the sclerotium of *Claviceps purpurea* (Fr.) Tul. (Clavicipitaceae) was used as an abortifacient in Central European folk medicine when Adam Lonicer (Lonicerus 1528–1586) described its application for the first time in 1582 (Ringrose, 1962). Women made use of repeated doses of three sclerotia against pains in the uterus (Lonicerus, 1962, p. 525). Prior to its acceptance as a birth-accelerating drug (thanks to the study by Stearns, 1808) authorities tried to ban ergot’s use (Ginzburg, 1990, p. 297). Indeed, ergot did not stand the test of time as a labor-inducing agent since the risks for the unborn were too high and, therefore, continued to be used only to stop the bleeding after parturition (Hofmann, 2002). The hemostatic principle of ergot was finally isolated, elucidated and named ergometrine in 1935 (Stoll, 1935). In an attempt to optimize the pharmacological characteristics of ergometrine Albert Hofmann (1906–2008) derivatized lysergic acid, the pharmacophore common to all ergot alkaloids, with butanolamine (instead of propanolamine as in ergometrine) obtaining methylergometrine, a compound with more favorable qualities. Still today the 5-hydroxytryptamine (5-HT) antagonist Methergin® is prescribed worldwide in midwifery for its reliable uterus contracting and hemostatic action. Methysergide, another ergometrine derivative interacting with 5-HT receptors was introduced to the market as a prophylaxis of migraine headaches in 1961 (von Bruckhausen et al., 1993, p. 970).

After the success with methylergometrine and in search of a circulation and respiration activator, Hofmann synthesized one of the most controversial compounds ever produced by humans: lysergic acid diethylamide (LSD-25). LSD was introduced as a research tool in psychiatry and sold under the name of Delysid by Sandoz who propagated this strong entheogen as a psyche-decentralizing agent in analytical psychotherapy (Hofmann, 2002, pp. 52–55). During the late 1950s and the 1960s LSD was widely used in psychotherapy alongside insulin shock therapy and with its psyche opening quality served as a therapeutic alternative to the tranquilizers frequently used at that time (Sandison, 1959a,b). Despite of the controversy accompanying the recreational use of LSD, which is generally hold responsible for the decline of therapeutic research with this psychedelic, it was recently pointed out how the difficulties in establishing its efficacy through controlled clinical trials additionally contributed to LSD’s demise in experimental psychotherapy (Oram, 2012).

The next example focuses on the same compound class but on a completely different ethnological background. In the Florentine Codex (a description of cultural practices in post-conquest Mexico) Bernardino de Sahagún (1499–1590) refers to the indigenous use of mind-altering mushrooms. The Florentine Codex is also referred to as “The Historia General de las Cosas de Nueva España” (General History of the Things of New Spain; ca. 1545–1590) and considered being the first truly ethnographic study due to the adopted methodological approach. In book 11 p. 130 Sahagún reports on inebriating mushrooms: “It is called *teonanacatl*.” It grows on the plains, in the grass. The head is small and round, the stem ling and slender…it makes one besotted; it deranges one, troubles one. It is a remedy for fever and gout. Only two or three can be eaten…. He who eats many of them sees many things, which make him afraid, or make him laugh. He flees, hangs himself, hurls himself from a cliff, cries out, takes fright. One eats it in honey…of one who is haughty, presumptuous, vain, of him it is said: “he mushrooms himself” (de Sahagún, 1961, Book 11, p. 130). Although Sahagún’s descriptions are quite precise it was not until July 1938 when the anthropologists Bassett Johnson (1915–1944) together with his wife and two colleagues were invited to attend a ceremony in Huautla de Jimenez (Oaxaca, Mexico) that the mushroom rite was rediscovered (Johnson, 1939; Wasson and Wasson, 1957, p. 237). In the same year Richard Evans Schultes (1915–2001) obtained fresh mushroom samples in Huautla, which he identified as *Panaeolus campanulatus* L. var. *sphinctrinus* (Fr.) Bresadola (Wasson and Wasson, 1957, p. 237). The first outsider to experience the effect of psilocybin containing mushrooms was Gordon Wasson (1898–1986) who in 1953 participated in a mushroom ceremony held by the *curandera* Maria Sabina in Huautla. His subsequent report “Seeking the Magic Mushrooms” published in Life Magazine brought this ancient and local knowledge to a broad North-American audience and assured that the mushroom rite received world-wide publicity. It was again Albert Hofmann who in the late 1950s isolated and elucidated the psychoactive principles of *teonanacatl*, the serotonin mimicking psilocin and its pro-drug psilocybin, disenchanting the magic mushrooms (Hofmann, 2002, pp. 118–126).

Both psilocybin containing mushrooms and LSD were soon experimented with outside the scientific community and became globally used recreational drugs influencing subcultures and propelling countercultural movements. The triggering of overwhelming psychedelic experiences led to the theory that the administration of the classical hallucinogens would result in an overall enhancement of brain activity in humans. This theory was relatively well accepted until recently although no thorough pharmaco-physiological studies of hallucinogen action have taken place since the 1960s (Lee and Roth, 2012). Intriguingly, a new study by Carhart-Harris et al. (2012) provide evidence that psilocybin leads to a decrease of cerebral blood flow and oxygen levels suggesting that the subjective cognition is caused by a reduced activity and decreased connectivity of the brain’s connector hubs (Carhart-Harris et al., 2012). In an interesting survey subjective case studies on cluster headache self-treatment with psilocybin and LSD were gathered (Sewell et al., 2006). Cluster headache is an extremely painful condition for which currently no medications terminating cluster periods or extending remission periods are available. Although the survey’s limitation due to non-clinical settings and an intrinsic selection bias, the results are intriguing, also because the apparent effectiveness of sub-hallucinogenic doses. A prophylactic effect of psilocybin perceived as complete cessation of attacks was reported by 52% of psilocybin users, while a further 41% reported a decreasing intensity and frequency of cluster headache attacks. Respondents also reported extension of remission period upon LSD and psilocybin consumption (see Sewell et al., 2006). New insights into the pharmaco-physiological interactions of hallucinogens and surveys conducted with individual users might help for a more directed and concerted use of psychedelic compounds in psychotherapy and pain therapy.

In the biogeographic study “On the trail of the ancient opium poppy” Merlin (1984) traces the cultural expansion of one of the most important and potent drug plants. Today opium alkaloids are a worldwide therapeutic commodity, while their molecular scaffolds inspired scores of pharmaceutical chemists and pharmacologists alike. One of the major concerns of medical opioid administration is the fast development of tolerance and withdrawal symptoms as well as minor complications such as constipation, emesis, and drowsiness (Darvishzadeh-Mahani et al., 2012). Recent experimental research on the therapeutic value of opioids (remifentanil) shows that single but highly dosed opioid administration reverses hyperalgesia in rats (Drdla-Schutting et al., 2012). This finding suggests that in case of hyperalgesia, instead of chronic low dose opioid administration, brief but highly dosed opioids might erase chronic pain persistently (Drdla-Schutting et al., 2012).

Also ginger (*Zingiber officinale* Roscoe, Zingiberaceae) besides its status as a spice and food item, has become a global medicinal commodity used against rheumatism, headache and above all against digestive and respiratory problems showing potent anti-inflammatory properties *in vitro* and *in vivo* (Chevallier, 2000; Williamson, 2002; van Breemen et al., 2011). While paradol, was found to inhibit COX-1 (Nurtjahja-Tjendraputra et al., 2003) 10-gingerol, 8-shogaol and 10-shogaol were shown to inhibit COX-2 (van Breemen et al., 2011) but shogaols and gingerols furthermore concurrently inhibit IL-1β and prostanoid secretion leading to strong upstream anti-inflammatory effects (Nievergelt et al., 2011). Murine *in vivo* data suggest that ginger extract administration is able to prevent morphine tolerance as well as naloxone-induced withdrawal signs (Darvishzadeh-Mahani et al., 2012). As an underlying mechanism L-type channel blocking properties by ginger constituents leading to an attenuation of calcium channel over expression induced by morphine has been suggested (Darvishzadeh-Mahani et al., 2012). The therapeutic benefit of ginger in this context might include its anti-inflammatory potential as it was shown that morphine triggers neuroinflammation in the CNS mediated via binding to the Toll-like receptor 4 (Wang et al., 2012b). Well-conducted clinical trials on the therapeutic efficacy and effectiveness of ginger preparations in painful inflammatory conditions are, however, still lacking (Terry et al., 2011).

*Bacopa monnieri* (L.) Pennell (Scrophulariaceae) is an important Ayurvedic medicinal plant used for a variety of health problems but above all to treat mental problems (Williamson, 2002; Russo and Borrelli, 2005). Since its Hindu name “Brahmi” is also valid for the Apiaceae species *Centella asiatica* L. (Sanskrit: Mandukaparni) the first written accounts are difficult to assign to either of the two species (see Hoernle, 2011, p. 301). Phytochemical analyses with *B. monnieri* started as early as 1931 and two saponins, bacoside A and B, are considered to be responsible for the neuropharmacological effects, while clinical trials with standardized extracts have shown promising results (Russo and Borrelli, 2005). Recent experimental data obtained with rats suggests that *B. monnieri* leaf extract up-regulates expression of tryptophan hydroxylase and serotonin transporters (Charles et al., 2011). A randomized controlled trial over a long-term period with a standardized *B. monnieri* extract is currently assessing the cognitive performance of subjects and tries to find correlations with and between inflammation, oxidative stress and cardiovascular health (Stough et al., 2012).

Semisynthetic derivatives of artemisinin, obtained from the ancient Chinese medicinal herb “qing hao” (*Artemisia annua* L., Asteraceae) are used in combination therapies and are a mainstay in the treatment of malaria (Kumar and Clark, 2001, pp. 80–81; Hsu, 2006). Artemisinin is a highly bioactive sesquiterpene lactone containing a 1,2,4-trioxane pharmacophore and its possible molecular mode of action has caused some debate (O’Neill et al., 2010). Besides artemisinin’s anti-plasmodial activity also its anti-tumor activity has come into focus (Woerdenbag et al., 1993; Efferth, 2007). The same rationale proposed for artemisinin’s anti-plasmodial activity may explain the sensitivity of cancer cells toward artemisinin (Mercer et al., 2011). Recent evidence suggests that hemoglobin degradation products such as heme are activating artemisinin leading to carbon-centered radicals exerting oxidative stress in the parasite (Klonis et al., 2011; Mercer et al., 2011). Artemisinin derived anti-malarial drugs are, however, not easily accessible or affordable to a large portion of the malaria struck population (Kokwaro, 2009; Wright et al., 2010). Therefore, *A. annua* has been introduced to rural areas afflicted by malaria epidemics around the globe for self-medication purposes. Locally grown and carefully prepared and applied herbal tea of *A. annua* could present a reasonable and affordable alternative (Wright et al., 2010; van der Kooy and Verpoorte, 2011). When adopting the preparation methods described in ancient Chinese medicinal texts it was shown that only a fraction of pounded juice is needed with respect to the quantity of aqueous infusion in order to provide the required amount of artemisinin (Wright et al., 2010). However, also by applying the correct infusion method artemisinin extraction efficiencies up to 90% can be reached (van der Kooy and Verpoorte, 2011).

Intriguingly, a survey about the anti-malarial use of *A. annua* in Kenya and Uganda revealed that in the meantime a considerable part of the respondents use the herb also against HIV/AIDS (Willcox et al., 2011). Encouraged by this report Lubbe et al. (2012) showed that *A. annua* as well as *A. afra* Jacq ex Willd., which does, however, not contain artemisinin, exhibit potent anti-HIV activity *in vitro*. Although the identification of the responsible compound(s) as well as *in vivo* studies are still due, Lubbe et al. (2012) have pointed out how people adapt their pharmacopoeia to the emergence of new diseases and epidemics. The use of “magic mushrooms” and LSD to treat cluster headaches and the use of *A. annua* in rural areas of Africa to combat HIV infection highlights that serendipity and the perception of collateral properties in the search of new medical therapies still plays an important role. Especially during the appearance of new diseases, lack of financial resources and in cases where no effective treatment methods exist (Sewell et al., 2006; Willcox et al., 2011) ethnopharmacology may lead us to effective therapies. These cases also highlight that ethnopharmacological research is not dependent on “traditional” knowledge systems in the narrower sense but that there exist feedback loops wherein modern and post-modern knowledge systems are becoming more relevant.

## DIET AND ETHNOPHARMACOLOGY

Etkin and Ross (1991) were referring to the “extra-nutritive” aspect of food phytochemistry and associated pharmacology when they asked whether we should “set a place for diet in ethnopharmacology”. The evolutionary perspective of health and nutrition comprises the co-evolution of the food-medicine continuum of wild gathered and cultivated vegetables (Etkin, 2006; Lindeberg, 2010; Leonti, 2012). Especially wild gathered food plants are often reported in local and popular traditions to have pharmacologic activities and are frequently associated with beneficial effects on the gastrointestinal tract, the cardiovascular system and with diuretic properties (e.g., Pardo-de-Santayana et al., 2005; Rivera et al., 2005; Guarrera and Savo, 2013). But although domestication of staple crops has led to a reduction of allelochemicals (Johns, 1990, pp. 102–159; Etkin and Ross, 1991), regularly consumed food products may provide relevant concentrations of pharmacologically active plant metabolites.

The focus thus falls on pharmacological properties of allelochemicals present in food, i.e., the bioactivity of secondary metabolites that evolved upon ecological constraints such as herbivory and not on our physiologic requirement for vitamins, pigments (carotenoids, lycopene) and fatty acids, which is considered in nutrition sciences (cf. Lindeberg, 2010; Carrera-Bastos et al., 2011). Also, antioxidant *in vitro* activities exerted by polyphenol-rich extracts and polyphenolic compounds such as flavonoids are hardly translatable into *in vivo* conditions and thus fall not in the narrower focus of ethnopharmacology. Following evolutionary concepts it was pointed out that flavonoids did not evolve for the purpose of scavenging free radicals (Zhang et al., 2010b).

The human dependence on salt in regions far from the seashore is occasionally satisfied by the production of vegetable salts from plant ashes. The salt obtained from the Witoto “woman-plant” (*Bactris humilis* (Wallace) Buret, Arecaceae) showed a remarkably high content of zinc and sodium with respect to salts obtained from other plant ashes. Zinc is a microelement required by pregnant and lactating women, while sodium is important for the retention and formation of amniotic liquid and might explain why the Witoto refer to *B. humilis* as “woman-plant” (Echeverri and Román-Jitdutjaaño, 2011).

A more regular example is the use of highly toxic *Aconitum* as root vegetable in the region of Shaanxi (China; Kang et al., 2012). Before being consumed, tubers of cultivated *Aconitum carmichaelii* Debeaux are boiled for up to 10 h, leading to degradation products of the diterpene alkaloids, which are far less toxic (Kang et al., 2012). The input for this dish, which is only consumed during the cold season and considered a “functional food” is in any case remarkable and warrants an ethnopharmacological analysis. Apart from the nutritional factors the ancient Romans highly appreciated the medicinal properties of cabbage, which they used for interior and external ailments (Matthioli et al., 1967–1970, book 2, p. 498). The claim that the juice of kale leaves has gastroprotective effects, preventing the formation of ulcers has been experimentally assessed (Carvalho et al., 2011; Lemos et al., 2011). Aqueous and hydroalcoholic *Brassica oleracea* L. (Brassicaceae) extracts (50–100 mg/kg) were found to be as effective as the positive controls in preventing experimentally induced gastric lesions in murines.

Insufficiently validated health claims and aggressive marketing strategies may, however, lead to negative socio-economic impacts on the local level. It was for instance not possible to date to scientifically confirm the claimed health benefits of açai (*Euterpe oleracea* Mart., Arecaceae) that range from rapid weight loss, prevention of cardiovascular diseases and aging in general (Heinrich et al., 2011). After an initial boost of worldwide açai commercialization triggered by local and global media coverage (Heinrich et al., 2011) the global sales of açai berries are expected to drop leaving trade volume to other, scientifically better corroborated nutraceuticals. In such a scenario local Brazilian farmers would be left empty handed. Another fruit enjoying growing popularity on the global health food market is the Goji berry (*Lycium* spp., Solanaceae; Potterat, 2010). While Goji berry products are considered a tonic and the juice a longevity drink, no such claims have hitherto been corroborated by scientific data not to mention by clinical studies (Potterat, 2010).

A closer cooperation of ethnopharmacologists with the agriculture sectors in areas such as food production, nutrition and health could counteract such scientifically deregulated developments (Heywood, 2011). It remains to be seen, however, what the priorities of the agriculture sector and the nutraceuticals industry are; easy money or controlled and rational health claims?

Turmeric (*Curcuma longa* L., Zingiberaceae) is an important ingredient in Indian and Chinese cuisine and medicine. India is the biggest producer and consumer of turmeric, while the US imports of turmeric stem to the largest extent from India. With respect to crude botanical materials, where the product price increases dramatically along the value chain (producer to consumer) without receiving additional value (final product is unprocessed) products including turmeric generally receive added value through different processing methods (Booker et al., 2012). In the case of mere encapsulated turmeric, which confers a medicinal appearance to the end product a 10-fold price increment has been observed (Booker et al., 2012). The active metabolite, curcumin, is actually a mixture of the three preponderant diferuloylmethanes also used as a colouring agent in food industry and labeled “E 100.” A large body of *in vitro* and *in vivo* evidence has been accumulated confirming curcumin’s anti-inflammatory properties (Goel et al., 2008). Clinical studies evidenced curcumin’s therapeutic relevance with rheumatoid arthritis, psoriasis, inflammatory bowel disease, inflammatory eye disease, and kidney transplantation (White and Judkins, 2011). In practice, however, curcumin administration suffers from the molecule’s hydrophobic nature, which determines its poor absorption, rapid metabolism, and limited tissue distribution. Also therefore, curcumin is well tolerated and regarded as safe, while doses as high as 12.000 mg per day have resulted in no significant adverse effects (Lao et al., 2006; Anand et al., 2007; Hsu and Cheng, 2007). Bioavailability of curcumin is, however, not so much an issue with the gastrointestinal tissues, mucose membranes in general and skin lesions, which can be exposed directly to biologically active concentrations (Hsu and Cheng, 2007). But curcumin has been shown to act as an iron chelator able to interfere with iron metabolism (Jiao et al., 2009). In patients with marginal iron stores curcumin supplementation may therefore lead to or aggravate anemia (Jiao et al., 2009). A possible solution with the aim at overcoming curcumin’s poor bioavailability and enhancing its aqueous dispersion is nanoparticle-encapsulation (Bisht et al., 2007). A mixture of curcumin with piperine, an alkaloid obtained from pepper (*Piper nigrum* L. and *P. longum* L., Piperaceae) able to enhance curcumin’s bioavailability via the inhibition of hepatic and intestinal glucuronidation (Shoba et al., 1998) has already been released to the market. Other brands contain mixtures of curcumin and broccoli (*Brassica oleracea* var. *italica* Plenck, Brassicaceae) seed extract with up to 6% of detoxifying sulfhoraphane glucosinolates and are currently sold for 39.95 USD (end user price) a bottle containing 30 servings (see also: ).

By nature of its assignment a completely different perspective regarding curcumin’s use as a food dye has been adopted by the European Food Safety Authority (EFSA; EFSA, 2010). Even though several previous safety evaluations by a joint FAO/WHO expert committee on food additives from 1974 to 2004 have established that curcumin was acceptable for use in food, EFSA re-evaluated the use of curcumin eliminating concerns over genotoxicity and concluding that consumption up to an average daily intake of 3 mg/kg bodyweight is safe (EFSA, 2010). This example highlights how different aims and perspectives generate disparate hypothesis, although EFSA’s concerns were primarily the chronic exposure to curcumin.

Recent commercial endeavors by the nutraceutical industry reveal a trend toward sophisticated formulations adopting the concept of polypharmacology or “network pharmacology.” Instead of approaching pharmacology with the concept of “one lock” (target) and “one key” (drug) Hopkins (2007) argued that one should consider that there are drug target networks and not just single disconnected drug targets. In herbal combination formulae practiced, for example in Ayurvedic and in Chinese medicine the pharmacokinetics and – dynamics of a single or a couple of compounds may be influenced by the rest of the metabolites present in the extract (Aggarwal et al., 2011; He et al., 2012, see Challenges in Ethnopharmacology). Medicinal plants have, however, not been “designed” to cure human diseases or alleviating their symptoms and undesired antagonistic effects are equally realistic. Re-engineer botanical drugs by designing extracts driven by therapy success could be a way forward toward “more intelligent mixtures” (Gertsch, 2011). The strategy of polypharmacology is as old as medicine itself and the food supplement industry provides several examples where phytochemicals of different vegetables, fruits or spices are blended to provide synergistic servings appearing as “molecular novel cuisine.” One should, however, keep in mind, that a variegated and balanced diet not only tastes better than pills and capsules, but is also healthier.

## DIACHRONIC STUDIES AND BIOPROSPECTING ANCIENT TEXTS

Since culture is not static but in a constant flux the importance of *materia medica* is exposed to different socio-economic factors and may change over time. These factors comprise cultural interaction (e.g., migration), change in diseases prevalence, lack or prove of efficacy and effectiveness, introduction of therapeutic alternatives (e.g., biomedicine), change in availability of new species and products and ecology (e.g., agricultural practices). Changes in medicinal plant use can be perceived as “continuity” (maintenance), “disjunction” (same species but new context or conceptual background), “discontinuity” (abandonment) and synchronism (substitution and adoption; see Bye et al., 1995). Odonne et al. (2011) found only one out of five previously described plant species used by the Wayãpi against leishmaniasis still in use, while previously not reported species are now relied upon. Generally field studies on medical ethnobotany show “snapshots,” i.e., the *status quo* of medicinal plant use for a certain community and point in time. Writing has enabled physicians, explorers, historians, and merchants to document, conserve and pass on gathered information and knowledge on medical plant use in codices, manuscripts and books. Thanks to these written sources plant uses can be traced diachronically through ages and over centuries up to the present usage (e.g., Leonti et al., 2010; Luczaj, 2010; Sõukand and Kalle, 2011; Obón et al., 2012; Pieroni et al., 2013) and even causal influences of herbal texts on modern local plant use can be observed (Leonti et al., 2010). Consequently, filtering down to the popular level, scientific literature influences local plant use and thereby shapes the results of current ethnobotanical field-studies. Quantitative diachronic evaluations of plant use additionally provide historic depth and information on the therapeutic rationales but of course would not be possible without “snapshots.” In the words of Steve Jobs (1955–2011) this approach reads “You can’t connect the dots looking forward; you can only connect them looking backwards” (Jobs, 2005).

Today, information regarding the use of medicinal and food plants is readily accessible via the WWW. While until recently global migration and urban environments were in the focus of changing and persisting plant use, the WWW increasingly strengthens such dynamics and renders them even more complex (Casselman and Heinrich, 2011). How the video-sharing platform YouTube^TM^ is disseminating and influencing global use patterns regarding the hallucinogenic application of *Salvia divinorum* Epling and Játiva (Lamiaceae) through user generated contents has been highlighted recently (Casselman and Heinrich, 2011).

In more economically developed countries and regions herbal medicine and phytotherapy is used as a therapeutic alternative to biomedicine for the treatment of mild and chronic health problems. Complementary and alternative therapies are also used to sustain conventional medicine and to treat their side effects such as the collateral symptoms frequently occurring during cancer therapy (Kronenberg et al., 2005). Moreover, today, a part of the world’s population has the choice of using herbal medicine (and also nutraceuticals) also as a fashionable life-style treatment. In many parts of the world conditions and opportunities for experimenting with herbal medicines in case of sever and acute health problems are not given anymore. Therefore, one question arising here relates to the differences in the application and function of for instance TCM or Ayurvedic medicine in rural and deprived societies with respect to a more prospering population.

During the pre-biomedical era, the use of herbal medicine and *materia medica*, together with primordial surgery and healing ceremonies were the only form of medical treatment. Historical texts documenting the ancient use of *materia medica* contain information, which is not present anymore in local knowledge of communities pertaining to industrialized nations. Therefore, ancient herbals may reveal interesting information for bioprospecting attempts. Buenz et al. (2004) provide some practical and theoretical considerations for approaching historic texts on *materia medica* as a source for drug discovery. Plant and drug identification in ancient texts can be challenging especially when a wide range of names is used for the same product and when no images are available (Lev, 2007). Another crucial point relates to the correct interpretation of the description of diseases, ailments, and the respective etiologies. When iconographical studies together with textual interpretations are possible (e.g., the case for most Renaissance herbals) the identification of most *materia medica*, is relatively straightforward, especially to the genus level. More complicated may be the correct interpretation of descriptions of amorphous products such as plant exudates (Lardos et al., 2011).

Malaria, for instance, was widespread in Europe and only eradicated toward the mid-twentieth century. The European Renaissance herbals make clear distinctions between remedies against *malaria tertiana* (*Plasmodium vivax*), and *malaria quartana* (*Plasmodium malariae*; Adams et al., 2011a). However, published data regarding their anti-plasmodial *in vitro* activity exist only for the minority of the taxa indicated against malaria in eight different German Renaissance herbals (Zimmermann et al., 2012). A comparative screening involving extracts obtained from species indicated against malaria and a control group of European medicinal species with indications other than malaria showed nine out of the ten most active extracts deriving from species with a documented anti-malarial use (Zimmermann et al., 2012). From frog’s spoon (*Alisma plantago-aquatica* L., Alismataceae), which is described in different sixteenth and seventeenth century herbals against “three” and “four day” fevers, protostane triterpenoids with moderate anti-plasmodial *in vitro* activity were isolated (Adams et al., 2011b).

## EVOLUTIONARY ASPECTS OF PHARMACOPOEIAS AND BIOPROSPECTING WITH PHYLOGENETIC APPROACHES

While drug discovery from natural products focuses on pure bioactive and bio-available, “drug-able” chemical entities, herbal medicine and phytotherapy research pursues the characterization of more complex pharmacological interactions exerted by an extract containing diverse chemical entities. The historical legacy testifies that ethnopharmacology has contributed to both domains, drug discovery and the development of herbal medicines (Elvin-Lewis, 2001; Fabricant and Farnsworth, 2001). Both domains focus on secondary metabolites, adaptive traits that evolved in response to ecological pressures. The fact that closely related taxa show more similar patterns of secondary metabolites than phylogenetically distant taxa, can be exploited for chemotaxonomical approaches in drug discovery.

A recent census regarding the taxonomic source of FDA approved and clinical-trial natural product drugs has provided evidence for a non-random distribution with clustered and disjunct taxonomic affiliations (Zhu et al., 2011). From a total of 740 existing Viridiplantae families (comprising ca. 450 Angiosperm families) only 59 families of the Angiospermae, four of the Gymnospermae, three of the mosses and one of the algae family provide all FDA approved and clinical-trial natural product drugs derived from plants (Zhu et al., 2011). Many of the Angio- and Gymnosperm species censed by Zhu et al. (2011; S5–S7) are also elements of local ethnopharmacopoeias and used in global herbal medicine or are used as spices and staple food and derive from an intimate human-plant relationship and have a history of an ethnopharmacological use. The “traditional” indications of the herbal medicines are, however, far more diverse and not always congruent with the biomedical applications of the purified and FDA approved compounds (Leonti et al., 2013). Similarly to FDA approved drugs, also the compositions of ethnopharmacopoeias show disproportionate selections of the available flora. Several statistical studies have pointed out that certain plant families (e.g., Asteraceae, Lamiaceae) are generally over proportionally relied upon for a selection within indigenous and local pharmacopoeias, while others (e.g., Poaceae and Orchidaceae) are normally a neglected source of medicine (e.g., Moerman et al., 1999; Saslis-Lagoudakis et al., 2011; Thomas et al., 2011; Weckerle et al., 2011; Weidenhammer et al., 2011; Turi and Murch, 2013). Most of these studies focus on ecological and universal aspects of herbal medicine and the different factors influencing plant selection, or advocate such statistical approaches as bioprospecting tools. A more sophisticated approach toward bioprospecting with comparisons of medicinal floras uses reconstructed phylogenies of ethnopharmacopoeias (Saslis-Lagoudakis et al., 2012). Exactly which practical instructions for bioprospecting purposes might be obtained from cross-cultural comparisons, or the analysis of disproportionate inclusion of plant taxa in pharmacopoeias, without considering the influence of other parameters, have both been put up for discussion (Gertsch, 2012).

Herbal medicines usually work with their entire bio-available chemical profile in synergistic and antagonistic relationships, which exacerbates bioprospecting attempts. Moreover, factors influencing medicinal plant selection by human communities are manifold ranging from ecology, cultural history, human cognition, and medical anthropology. Recently the first author characterized the prototypical medicinal plant as “an abundant Euasterid weedy species, possibly utilizable as food, routinely attracting pollinators, rich in pharmacologically active and bio-available metabolites, endowed with pronounced organoleptic properties and ideally, with a bit of fantasy, the signature of the organoleptic virtues could be matched with the plant’s medicinal indication, which thereby would facilitate its transmission and through the belief in its semiotic appearance at the same time enhance the meaning response of its medical application” (Leonti, 2011). But one of the crucial points with respect to drug discovery is that most medicinal species are used for a wide range of therapeutic applications, which renders the formulation of tangible instructions for bioprospecting attempts difficult. It has also been shown that when indigenous medicines are evaluated across a range of bioassays, ethnomedical indications rarely correspond with meaningful screening results (Gyllenhaal et al., 2012). A particularly interesting fact is, however, that plant species used as medicine by human societies achieve higher number of hits with respect to random collections when screened in cell based and mechanistic bioassays (Spjut and Perdue, 1976; Gyllenhaal et al., 2012).

This may be due to the overrepresentation of weeds and widespread taxa in local and official pharmacopoeias (Stepp and Moerman, 2001; Stepp, 2004); it has been proposed that taxa with wide biogeographical distributions have encoded a broader range of ecological responses in their genes with respect to more locally occurring taxa (Leonti et al., 2013). These responses include the synthesis of allelochemicals with broad-spectrum biological interactions, comprising the targeting of proteins of mammals and primates.

## MEANING RESPONSE, EFFICACY, AND EFFECTIVENESS

With respect to biomedicine, traditional systems of medicine base diagnostics, perception of illness and healing traditions on elaborate, and from a Western scientific point of view, challenging (if not unacceptable) philosophy (e.g., Jiang et al., 2012; Mukherjee et al., 2012; see Foster and Anderson, 1978). Often these philosophies are grounded on physiologic equilibrium models, which are also expressed through curing rites and psychosocial support (Foster and Anderson, 1978). Such philosophies and holistic approaches comfort the end-user and thereby impart a beneficial and self-affirming “placebo effect” also called “meaning response” (cf. Moerman and Jonas, 2002). Higher expectation on treatment outcome by patients with chronic pain had a significant impact on the effectiveness of acupuncture therapy (Linde et al., 2007). It has been suggested that psychological factors such as expectation and behavioral conditioning are able to induce neurobiological mechanisms that interact with pharmacological pathways (Bingel et al., 2011; Rief et al., 2011). Although the pharmacological assessment and evaluation of the meaning response goes beyond capabilities of standard pharmacology laboratories, the relevance of the placebo and the nocebo effect may not be recognized enough: the therapeutic context, in which an optimal drug efficiency may be achieved should be included in the treatment recommendations (Rief et al., 2011). Even though 25 years ago Etkin (1988) already pointed out how drug efficacy depends on the specific socio-cultural context, this insight has not been incorporated widely into clinical studies to the extent it might deserve (Witt, 2013). Last et al. (2011) defined “efficacy” as treatment outcomes obtained under ideal and standardized conditions (e.g., clinical study setting) as opposed to “effectiveness,” which is used to describe the therapeutic success in routine and non-experimental circumstances (see also Witt, 2013). As globalization changes socio-cultural contexts one can expect that the current development will have an impact on the meaning response of a specific therapy. For example in Western societies “more” is regarded as “better” above all in relation to monetary affairs. One plausible hypothesis would therefore be that more sophisticated and costly a (herbal) preparations would achieve more pronounced placebo effects, and hence effectiveness, also on the global scale.

## TRADITIONAL MEDICINES: GLOBALIZATION, KNOWLEDGE TRANSMISSION, INTEGRATION, AND MEDICAL PLURALISM

For the largest part of human history information and knowledge has been transmitted orally and by observation and direct copying. With the advent of writing systems a more precise copying and knowledge transmission was possible and thanks to scripts the transmission of knowledge related to *material medica* over wide ranges of space and time was possible. Oral and written knowledge transmission is in constant exchange and written knowledge may result in changes to traditions, which are then passed on orally – and *vice versa* – and from that point of view a dichotomization makes no sense (Totelin, 2009). However, there exists a clear quantitative difference in knowledge transmission emanated by texts used as guides and references over a long period of time, over broad geographical extensions and diverse cultural backgrounds. Generally, scripts allow a more conservative knowledge transmission and may lead to a homogenization of knowledge (Leonti, 2011).

That goods and associated knowledge from Indian and Chinese *materia medica* were exchanged along the Silk Road reaching and influencing early Mediterranean medical traditions can be traced in scripts dealing with *materia medica* at least from the fifth to the fourth century BC onward (Touwaide and Appetiti, 2013). On the other hand received the Hindu system of medicine at the same time apparently little external influences. A collection of 51 birch-bark leaves known as the Bower Manuscript is regarded as the oldest still existing script dealing with Hindu medicine (Winternitz, 1902). It was discovered in Kucha (Chinese Turkestan) situated on the Silk Road in 1889, translated by the German-British orientalist Rudolf Hoernle (1841–1918) and dated to the fifth century AD. Not even opium, which at that time was one of the most important drugs in the Mediterranean and the Near East, is mentioned in the Bower Manuscript (Tschirch, 1910, p. 508; Hoernle, 2011). Although earlier, and now lost, medicinal scripts dealing with ancient Hindu medicine existed, such medical knowledge within India has mainly been passed on by practitioners and through the individual training of pupils (Srivastava, 1954; Foster and Anderson, 1978, p. 62; Mukherjee et al., 2012). Medicinal practices on the Indian subcontinent began to intermingle with the Arab system of medicine (Unani Tibb) from the twelfth century onward and toward the end of the eighteenth century the British began to introduce the Western system of medicine (Srivastava, 1954). The first European medical school in India, the Calcutta Medical College, opened in 1835 (Anonymous, 1879). This historical development led to the current situation that, while the Sanskrit classics altogether describe some 700 medicinal plant species, depending on the source, more than 10,000 plant species are associated with Ayurvedic medicine (Padma, 2005).

**FIGURE 1:**
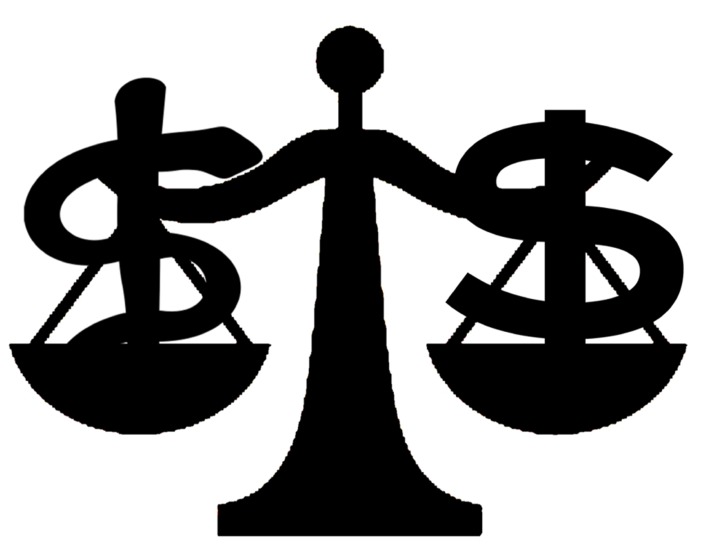
**A fair price for medicine – does it exist**?

Thanks to global commercialization and intercultural knowledge and information exchange, patients increasingly have the choice between different medicinal systems for their health care needs with access to physicians specialized within different medical systems (e.g., Miles, 1998; Khan, 2006; Stranieri and Vaughan, 2010). Based on the historical and economic development in a country or region a nation’s traditional system(s) of medicine is often complemented with biomedicine. Conversely, in other regions, alongside biomedicine different traditional systems of medicine and alternatives (complementary medicine) are established. If and how alternative therapies should be included and covered by national health services and what is a fair price for individual therapies, medicine and health care in general is the matter of continuous debates (**Figure 1**).

However, Khan (2006) argues that although medical pluralism is already a historic reality in many societies there is a need to bring the discussion beyond the pluralist and liberal arguments including factors such as “domination and hegemony” widening the historical, social and political context of the discussion. With respect to India, Khan (2006) specifies, that while Western culture and medicine exerted authority over indigenous science and medicine during the colonial phase it were the national leaderships and governments, which consolidated this development. On the other hand did the demand for indigenous alternatives, according to Khan (2006), although effective, not get enough response from the national authorities. Likewise, local health care and medicine is subjected to global commercialism, which progressively influences individual therapeutic choices and preferences (cf. Miles, 1998). But while patients may choose between different medicinal systems, therapeutic settings, where specialists from different medicinal systems collaborate, are rare. One recently highlighted example stems from a modern community hospital in Thailand, where Thai Traditional Medicine is integrated alongside biomedicine into the modern healthcare service (Chotchoungchatchai et al., 2012). The identified key factors for the successful integration are knowledge transmission of Royal Thai Traditional Medicine and locally practiced herbal medicine to the hospital practitioners and a healthcare team consisting of members with different backgrounds, together with a well organized supply of herbal medicine (Chotchoungchatchai et al., 2012). Integrating medical systems is not only a challenge for hospital facilities but also in health informatics (Health Information Systems), which is currently closely linked to the dimensions of Western biomedicine (Stranieri and Vaughan, 2010). Since the trend toward co-existence of different medicinal systems is bound to augment it is appropriate to design health informatics applications compliant with the characteristics of different medicinal systems. The visualization of arguments *pro* and *contra* for choosing a certain medicinal system in a particular health condition is put forward by Stranieri and Vaughan (2010) as an example of how the coalescing of medicinal systems into health informatics may support patients’ informed decisions making. In this respect Witt (2013) argues that there is a lack of clinical studies focusing on comparative effectiveness of traditional herbal medicine and food products.

Traditional medicines will only be taken seriously and accepted by primary health care systems when experimental and clinical data establish an evidence base (Cordell, 2011; Verpoorte, 2012). High standard studies confirming the safety and the effectiveness of traditional medicines are, therefore, required (Cordell, 2011; Verpoorte, 2012). Since co-medication of medicinal plants and biomedicine is frequently observed, practitioners and clinicians are encouraged to watch out for adverse as well as beneficial interactions (Giovannini et al., 2011; Nagata et al., 2011; Odonne et al., 2011). Clinical studies instead of focusing exclusively on efficacy could focus also on effectiveness by including a more heterogeneous sample of participants and adopting research settings that reflect usual heath care situations as far as possible (Witt, 2013). According to Verpoorte (2012) and others (see Weidenhammer et al., 2011 and Xu et al., 2013), the changing global economic scene and changing paradigm in drug development might present an advantage in the attempt to officially register traditional medicines with governmental agencies. However, as pronounced by Etkin (2006, p. 212), “aggressive marketing of both pharmaceuticals and supplements blurs the line between scientific observation and product advertisement.”

## CONCLUSION

The use of indigenous drugs, herbal medicine and traditional *materia medica* can only be understood through a combination of historical, ecological, economic, cognitive, and pharmacological approaches, while anecdotal references are lost in space and time. Ongoing globalization driven by neo-liberalism increases medical plurality through intercultural knowledge and information exchange. Although there is a trend toward the development of global pharmacopoeias, local pharmacopoeias, despite being influenced by global economy, and politics will continue to keep their role and identity. While knowledge transmission along trade and value chains tend to secure hegemonic and financial interests over local economies and pharmacopoeias, the local availability of biomedicine and access to research data on traditional and herbal medicine may lead to a syncretic development with reconciling effects. Not only herbal products but also food supplements and nutraceuticals have considerably raised their market share in the last few years. Since sound scientific data for many commercialized health products is still lacking, this calls for a much more rigorous multidisciplinary science-driven approach to local and traditional medicines, which also empowers the local keepers of this knowledge and their users.

## Conflict of Interest Statement

The authors declare that the research was conducted in the absence of any commercial or financial relationships that could be construed as a potential conflict of interest.
